# Zinc therapy for acute diarrhea in children under three years of age

**DOI:** 10.1186/1471-2334-14-S7-P80

**Published:** 2014-10-15

**Authors:** Nicoleta Negruț

**Affiliations:** 1 Faculty of Medicine and Pharmacy, University of Oradea, Romania

## Background

Zinc therapy is recommended by WHO and UNICEF in acute diarrhea in children under 5 years old.

## Methods

The study’s outcome was to investigate the efficacy of the zinc treatment in children aged 0-3 years, from the region of Bihor, Romania, with acute diarrhea. Zinc sulfate (10-20 mg daily, according to age) was given to the patients in the study group for 10 days. All children were followed up over a period of 3 months for new episodes of diarrhea. Data were processed using IBM SPSS statistics version 22.

## Results

During 2009-2011, 116 children with acute diarrhea from Bihor country, Romania, were enrolled in the study. From initial group, 103 children were available for final analysis. The duration of diarrhea in the study group (n=53) as compared to control (n=50) was reduced by 24 hours (1.94±0.7 days versus 2.4±1.2 days, p <0.001, Student’s t test). Diarrheic episodes in the next three months occurred more frequently in the control group (3.8% versus 16%, p=0.036, Student’s t test).

## Conclusion

Zinc supplementation has a significant effect on the duration of acute diarrhea. The mineral can reduce the frequency of acute diarrhea in the next 3 months.

